# Pathogenic Bacteria in Free-Living Birds, and Its Public Health Significance

**DOI:** 10.3390/ani14060968

**Published:** 2024-03-20

**Authors:** Aleksandra Kobuszewska, Beata Wysok

**Affiliations:** Department of Veterinary Public Health, Faculty of Veterinary Medicine, University of Warmia and Mazury in Olsztyn, Oczapowskiego 14, 10-710 Olsztyn, Poland; beata.wysok@uwm.edu.pl

**Keywords:** wild birds, antimicrobial resistance, pathogen transmission, migratory species, zoonotic diseases

## Abstract

**Simple Summary:**

The avian population, occupying a diverse range of ecological niches and urban environments, serves as a crucial reservoir and sentinel for various pathogens. The role of birds in the transmission of infectious diseases is well established, and their ability to harbor a wide range of pathogens has been documented by numerous studies. As such, their surveillance and monitoring can provide valuable insights into the emergence and spread of bacterial diseases. Therefore, understanding the complex interactions between birds and their pathogens is of utmost importance in the field of public health and requires continued research and investigation.

**Abstract:**

Birds that roam freely, particularly those that migrate, have the potential to carry a range of diseases that can be passed on to humans. The vast movement of these birds across diverse environments and urban areas can contribute to the spread of bacteria over long distances, impacting both human and animal populations. Stress, overcrowding, and human interaction can also play a role in the transmission of infectious diseases among birds and humans. Therefore, it is crucial to comprehend the intricate connections between birds, vectors, zoonotic pathogens, and the environment, especially given the increasing urbanization and emergence of zoonotic illnesses. This review aims to provide a systematic overview of the significance of avian species in transmitting bacterial pathogens that pose a risk to public health.

## 1. Introduction

Free-living birds are perceived as important sources of viruses, bacteria, and fungi that can significantly impact other animal populations and human health. Given their large movement capacities, they might transfer numerous pathogens during migrations, which could result in intra- and interspecies transmission [1]. This creates the potential for the establishment of novel foci of emerging or re-emerging communicable diseases along bird migration routes [2]. Birds, in particular free-living birds, have been widely reported as reservoirs of pathogens and, due to their ability to occupy different ecological niches and to adapt to many urban, suburban, and livestock environments, represent true sentinels [3]. Numerous studies conducted worldwide have reported the presence of various bacteria in wild birds, many of which may have a high zoonotic potential [4,5,6]. Long-distance migrants, such as barn swallows, fly from Europe to Africa for wintering, covering a distance of over 10,000 km every year [7,8]. Researchers discovered that migrant swallows were considerably more likely than resident swallows to have *Salmonella* spp. in their microbiome [9], which might play a role in the national and international dissemination of this bacteria. Some factors may significantly determine the role of wild migratory birds in the spread of certain pathogens. 

The impact of stress levels and crowding on wild birds can lead to the transmission of infectious diseases among them, resulting in a rapid spread of pathogens throughout the population [2]. In light of their high population densities, close social interactions, spatial mobility, and capacity to colonize anthropogenic environments, birds possess significant potential to serve as reservoirs for pathogens that can be transmitted to other vertebrates, including humans [10]. Simultaneously, when people enter the habitats of wild birds, including to feed them, they become exposed to pathogens. It is important to note that birds, due to their mobility and the deposition of droppings, can act as vectors for the environmental circulation of zoonotic agents. Additionally, they may also spread antimicrobial-resistant bacteria with various resistance gene profiles, making it crucial to monitor their role in the spread of infectious diseases [11].

When raising the subject of wildlife fowl, the microbiological risks from the production and consumption of game birds cannot be omitted. They can carry common foodborne bacteria (e.g., *Salmonella*, *Campylobacter*, *Yersinia*, and *Listeria*) in their intestines [12,13]. Because game birds are generally asymptomatic carriers of pathogenic bacteria, these factors can easily be disseminated to hunters and consumers unnoticed through the handling and consumption of infected game birds [13]. However, the microbial contamination of game bird carcasses may be affected by factors like shot location, slaughter hygiene, and the cold chain.

Understanding the connections between birds, vectors, zoonotic pathogens, and the environment is crucial, given rising urbanization and the rise of zoonotic illnesses [14], as they comprise over 75% of all emerging human illnesses [15]. The objective of the present review is to evaluate the possible transmission of pathogenic bacteria from wild birds to the surrounding environment. These bacteria can then be transmitted to humans, highlighting the interconnection between environmental, animal, and human health. It is worth noting that most birds infected with zoonotic bacteria do not display clinical symptoms and are asymptomatic carriers. However, they pose a significant risk to human health, making it crucial to understand the potential dangers.

## 2. Materials and Methods

**Figure 1:**
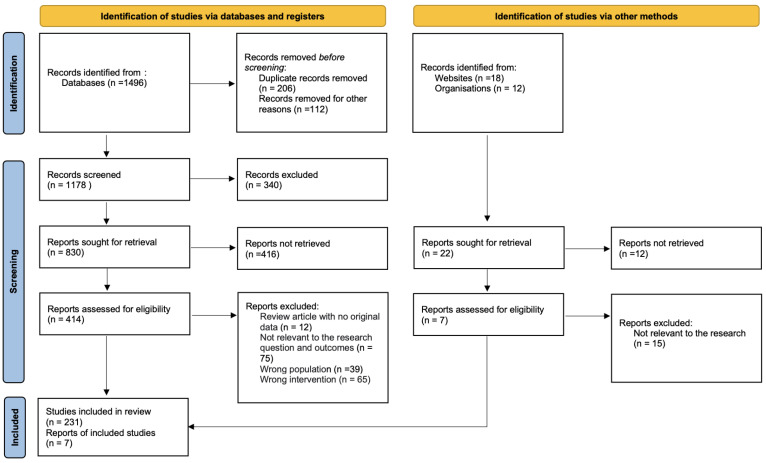
PRISMA flow diagram showing article selection, documenting included and excluded articles at each stage.

A systematic literature review was undertaken to elucidate the role of free-living birds as reservoirs and vectors of pathogenic bacteria, adhering to the Preferred Reporting Items for Systematic Reviews and Meta-Analyses (PRISMA) guidelines (Figure 1).

The search strategy encompassed electronic databases, including PubMed and ScienceDirect, to identify pertinent articles published from September 2022 to January 2024. Search terms were meticulously chosen to cover a broad spectrum of relevant topics, including variations of “free-living birds”, “wild birds”, “migratory birds”, “avian”, “pathogenic bacteria”, “bacterial pathogens”, “resistance”, “virulence genes”, and related terminologies.

A total of 238 articles were meticulously selected for inclusion in this review, based on their relevance and contribution to understanding the role of free-living birds in the dissemination of pathogenic bacteria. Systematic data extraction was conducted, focusing on key elements such as bacterial species identified, host species involved, geographical locations, transmission routes, and public health implications.

Selected articles underwent critical evaluation for methodological rigor. Parameters assessed included study design, sample size, sampling methods, laboratory techniques utilized for bacterial identification and characterization, and statistical analyses employed to ensure robustness in the findings.

Any limitations or biases identified within the included studies were meticulously noted and discussed within the context of this review, thereby providing a systematic analysis of the synthesized evidence.

## 3. Bacterial Pathogens in Wild Birds

Campylobacteriosis is the most frequent zoonotic disease reported worldwide [16]. *Campylobacter* spp. can colonize the intestinal tract of most mammals and birds, both domestic and free-living. Although these bacteria are able to colonize the intestines of both domestic and free-living birds, are generally perceived as non-pathogenic. This assumption has been confirmed by different histopathological investigations, which showed no pathological lesions or marked alteration in crypt features in birds in a commensal state [17]. Usually, transmission of *Campylobacter* from birds to humans is due to consumption of poultry products, as poultry carcasses undergo cross-contamination during post-slaughter processing, mainly during defeathering and evisceration. Meta-analysis performed by Ansarifar et al. [18] reveals that the quail meat has the highest contamination (68.4%) with *Campylobacter* spp. followed by chicken (56.1%), turkey (27.4%) and ostrich meat (11.7%). The frequency of *Campylobacter* in wild birds may be affected by several variables, including geographic location, season, the health of the bird, the species of bird, the type of sample taken, the methodology, and ecological factors [19]. There have been reports of *Campylobacter* spp. in a variety of wildlife species, including squab [20], quail [21], guinea fowl [22], and duck [23,24,25]. Generally, *Campylobacter* isolates recovered from wild birds are described as genetically divergent; however, despite the high genetic divergence among environmental *Campylobacter* isolates, overlapping of sequence types of isolates derived from human outbreaks and wild birds is still observed [26,27]. An investigation performed in 2008 including the analysis of *Campylobacter jejuni* isolates from human, raw pea, and wild bird faecal samples confirmed the epidemiologic link between illness and the consumption of raw peas contaminated by sandhill cranes [27]. In a study performed by Abdollahpour et al. [28], wild bird faeces were identified as a source of *Campylobacter jejuni* infection in children’s playgrounds in Iran. Children’s behaviour, such as frequent hand–mouth contact, contributed to the ingestion of birds’ faeces that carried pathogenic bacteria. On the other hand, studies performed by Southern et al. [29] and Stuart et al. [30] showed the correlation between *Campylobacter* infection in humans and drinking pasteurized milk from bottles with damaged tops due to attacks by birds.

The wide distribution of *Campylobacter* spp. among wild birds also poses a risk to the farm environment. In the study conducted by Sanad et al. [31] in the USA, genotypically identical isolates of *Campylobacter jejuni* were recovered from the faeces of starlings and cattle on the same establishment. These bacteria were detected in 36.6% of dairy and 50.4% of starling faecal samples, and several genotypes overlapped between dairy cattle and starling isolates. These findings point to starlings as possible *C. jejuni* reservoirs and their potential impact on the epidemiology of clinically significant *C. jejuni* in the dairy community. The common distribution of *Campylobacter* among free-living birds poses a specific risk in the case of species that can reach the consumer’s table as a by-product of the shooting industry. The studies conducted by Kovanen et al. [32] in Finland showed that game birds may pose a risk for acquiring campylobacteriosis because they had *C. jejuni* genotypes highly similar to human isolates detected previously. Therefore, hygienic measures during slaughter and meat handling warrant special attention.

*Listeria monocytogenes* is the causative agent of human listeriosis, a severe illness that is particularly fatal for the elderly, pregnant women, and newborns [33]. It is a dangerous disease with a considerable fatality rate of 20–30% worldwide [34]. Listeriosis is a serious infection usually caused by ingestion of food contaminated with *L. monocytogenes*; however, birds may disseminate *L. monocytogenes* in nature and may also contaminate foods when entering food processing environments and outdoor marketplaces [35]. These bacteria are common in natural habitats like soil, surface water, sewage, or mammals’ and birds’ faeces [36,37]. However, many species of birds, including chickens, turkeys, pigeons, ducks, geese, pheasants, canaries, and cockatiels, are susceptible to natural infection, and clinical disease in birds is rare [38]. Birds can be infected via the airborne route by inhalation, uptake, or debeaking, and young birds appear to be more susceptible to *Listeria* infection than older birds, with listeriosis most commonly manifested as septicaemia [39]. The prevalence of this pathogen in wild birds varies from 3.4% in pigeons to 46% in urban rooks [35,40,41,42,43]. Hellstrom et al. [35] discovered that the incidence of *L. monocytogenes* in faeces is higher in birds at landfill sites than in metropolitan areas, and isolated strains are frequently similar to those detected in foods and food processing environments [35]. Widespread contamination of the environment with birds’ faeces can become a source of food- and feed-borne illnesses in humans and animals, with the chain of food production reflecting a continuum between the farm environment and human populations predisposed to listeriosis [44]. In 2013, an outbreak of listeriosis in a small pheasant breeder farm was described. Approximately 300 young birds and a few adult birds were found dead within a few days of the onset of clinical signs, which included diarrhoea and significant neurological symptoms. *L. monocytogenes* was observed in heart blood smears, liver, and brain impression smears [38]. This information is very alarming as the common pheasant is the most hunted, widespread, and economically important non-migratory game bird in Europe [45].

*Clostridium perfringens* is one of the major causative agents of enteric diseases both in humans and animals [46]. The main sources of this bacterium are healthy birds’ digestive contents, soil, dust, poultry litter, and animal faeces [47]. *C. perfringens* has been isolated from many bird species, such as ducks [48,49], wild crows [50], western bluebirds [51], ostriches [52], greater sage-grouses [53], pheasants [54], and capercaillies [55]. Moreover, once infected, the birds can transmit pathogens by dropping contaminated feces, which becomes a significant risk factor for illness among individuals and domestic animals. The study conducted by Craven et al. [56] revealed the prevalence of this enteropathogen in wild birds’ faeces near broiler chicken houses, which suggests that wild birds that gain entry to poultry grow-out houses have the potential to transmit these pathogens to poultry.

Necrotic enteritis (NE) caused by *C. perfingens* results in huge economic harm to chicken farms [57], as these bacteria that cause clinical or subclinical necrotic enteritis have the ability to synthesize toxins, bacteriocins, and enzymes of different natures, which modify the anatomical structure of the intestinal mucosa, enterocytes, and the cellular matrix, altering the physiological activities of the gastrointestinal tract, resulting in gastrointestinal disorders and diarrhea [47]. Seki et al. [58] examined the isolation and toxinotyping of *C. perfringens* from wild crows, starlings, and sparrows to clarify the relationships of these resident birds with the incursion of this bacteria to poultry farms. Among the resident birds examined, *C*. *perfringens* was isolated from 51.3% of large-billed crows, 43.4% of carrion crows, and 28.0% of white-cheeked starlings. As a result, it was suspected that among wild bird species subjected to testing, crows were likely to have a relationship with the incursion of *C. perfringens* to poultry farms.

Another significant multi-host pathogen is *Staphylococcus aureus*, a commensal organism that is able to effectively colonize a wide variety of host species, including many mammalian species and also birds [59]. This pathogen is part of the normal flora of the skin and other mucous membranes of poultry; however, in birds with reduced immunity, after crossing physical barriers, it gains access to tissues and the bloodstream and may cause local or systemic infection of poultry. *S. aureus* may cause septicaemia, fibrinous arthritis and tenosynovitis, chondronecrosis, and osteomyelitis in chickens, turkeys, ducks, and geese [60]. Staphylococcal food-borne disease (SFD) is one of the most common foodborne diseases worldwide, resulting from the contamination of meat and meat products, even though, when it comes to poultry consumption, Staphylococcal food-borne disease has mostly been linked to the ingestion of chicken meat [61,62,63,64]. Wide distribution of *S. aureus* among wild birds also was observed, this pathogen was isolated from birds of prey (33% on average) [65,66,67], lesser yellowlegs (0.87%) [68], geese (7.1%) [69], magpies, cinereous vultures (8.3%) [70], white storks (34.8%) [71], and white ibis (10.9%) [72]. As Sousa et al. [65] underlined, birds seem to be a natural reservoir of *S. aureus* and coagulase-negative staphylococci resistant to several antibiotics. Interaction between wildlife and people is becoming more frequent as habitats converge, implying an increasing chance of exchange of these microbes in the various ecosystems. Raptors such as owls that feed mostly on small mammals and insects and are in direct contact with many species of wild rodents operate as a significant vector for the transmission of pathogens between humans and animals [66]. Some researchers suggest that wild birds’ droppings may also contribute to the contamination of bathing water with *S. aureus* [73]. According to Cragg and Clayton [74], *S. aureus* is one of the most prevalent bacteria in seagulls’ faecal flora; hence, beaches with more seagulls may be considered as at a higher risk of *S. aureus* contamination. Generally, Staphylococci have been identified as an appropriate model for “One Health” investigations, as some species and clones have been demonstrated to be bound through the three ecosystems of interest (humans, animals, and the environment) [75].

*Escherichia coli* is a normal commensal in the guts of birds and animals; however, some wild birds may carry pathogenic strains of *E. coli*. Likewise, enteropathogenic *E. coli* (EPEC) and Shiga-toxin-producing *E. coli* (STEC) [76] belong to the category of diarrheagenic *E. coli* that can cause serious disease in humans [77]. The EPEC pathotype leads to a high child mortality rate in developing countries, and diarrhea caused by these strains is a consequence of loss of intestinal microvillus, while STEC infection causes haemorrhagic colitis following the injury of the intestinal epithelium [78]. In Italy, STEC was isolated from wild ducks and live common quail faecal matter, which indicates the potential role of migratory ducks and quails in the transmission of STEC [79]. A study performed in Brazil by Sanches et al. [80] proved that captive wild birds are reservoirs of enteropathogenic *E. coli* and Shiga-toxin-producing *E. coli* strains. In that study, faecal samples were analysed from 516 birds in two municipal zoos located in São Paulo State, Brazil. A total of 401 isolates were identified as *E. coli.* The pathotype classification of the isolates showed that typical EPEC was detected in 2.99%, atypical EPEC in 1.99%, and STEC in 0.74% of samples. Even though the frequency of these pathotypes was low and restricted to a few orders, the data suggest the potential public health risk that these birds represent as reservoirs of diarrheagenic *E. coli*. Borges et al. [77] isolated two STEC (0.8%) and five EPEC strains (2.0%) from wild birds, and four EPEC strains (2.0%) were from pigeons. The incidence of EPEC and STEC in wild birds was comparable to that reported in previous investigations of these free-living avian species, where rates varied from 0% to 1.8% for STEC strains [81,82,83,84,85] and from 2.8% to 4.9% for EPEC strains [83,86,87,88]. The authors underline that wild birds could act as carriers of STEC and EPEC and, therefore, may constitute a considerable hazard to human and animal health by transmission of these strains to the environment.

The intestines of wild birds are very often colonized by *Salmonella* spp., mostly with no signs of infection, which therefore can be shed in faeces. These bacteria have been found among various species, such as passerines (8.8%) [89,90,91,92], teals (3.3%) [13], pigeons (27.5%) [93,94], and guinea fowls (8.9%) [95]. According to Tizard [96], salmonellosis in wild birds occurs most commonly in those on a carnivorous or omnivorous diet, those that feed on the ground or on the food subject to faecal contamination, or those that live or feed in contaminated water. Although several serovars of *Salmonella* may be found in the intestines of wild birds, and they persist within bird populations through several mechanisms, the wild birds may be temporary or permanent *Salmonella* carriers or even suffer from clinical salmonellosis [96]. Several studies have confirmed mass die-offs of free-ranging birds of various taxa due to infection with *Salmonella* spp. [97], and it has been estimated that *Salmonella* spp. was responsible for 21.5% of passerine cases and 5.4% of total bird mortality in the United States in the years 1985–2004. The well-documented prevalence of *Salmonella* among free-living birds promotes the spread of this pathogen in the environment and poses a real threat to human health and life. The Centre for Disease Control and Prevention (CDC) confirmed an outbreak of salmonellosis in humans linked to songbirds, as they reported a total of 29 people infected, aged from 21 days to 89 years. Illnesses started on dates ranging from 26 December 2020 to 29 April 2021, in 12 states. Of the 21 people interviewed, 13 (62%) reported owning a bird feeder and 4 (19%) people reported contact with a sick or dead wild bird, while 15 people (71.4%) had pets that had access to or contact with wild birds [98]. Moreover, Lawson et al. [99] emphasize that garden birds are a source of human salmonellosis and highlight how important personal hygiene measures are when feeding wild birds. The majority of documented outbreaks of passerine salmonellosis happened at or around feeding stations, which are probably places where people might come into contact with ill or deceased garden birds and their droppings [99]. Additionally, Craven et al. [56] suggest that wild birds that gain entry to poultry grow-out houses have the potential to transmit these pathogens to poultry. On the other hand, wild birds can also pollute food that will be directly consumed by humans.

Free-living birds may also be noticed as a significant vector of pathogenic *Vibrio* spp. Around 12 of the roughly 100 species in the genus *Vibrio* are related to clinical illnesses, such as cholera and vibriosis [100]. Authors found that the burden of disease in birds was most associated with *V. cholerae*, followed by *V. metschnikovii* and *V. parahaemolyticus*. Prevalence of *Vibrio* spp. in birds varies from 43% in crows [101], and 48% in gulls, up to 65% in Magellanic penguins [102]. Researchers have discovered that migratory waterbirds can be a long-distance vector of pathogenic *Vibrio* species, which can be a potential threat to public health. These findings were confirmed by Fu et al. [103], who, using high-resolution genome sequencing, noticed *V. parahaemolyticus* strains belonging to the same clone in faeces samples derived from an area at a distance of over 1150 km. The role of migratory birds as *Vibrio* disease vectors was also described by Zheng et al. [104]. These authors found a significant level of variation across the strains tested, with numerous novel *V. cholerae* alleles among migratory birds that had not previously been identified in databases. In another study conducted in Romania by Pall et al. [101], a higher number of *Vibrio* strains in migratory (74.66%) than in sedentary birds (25.33%) were indicated, with increased risk of transmission to humans or the environment in places where they transit or nest.

The *Yersinia* genus is comprised of 28 distinct species, of which 3 have the potential to cause illness in humans. *Yersinia pestis* is the culprit behind the plague, while *Yersinia pseudotuberculosis* and *Y. enterocolitica* are enteropathogenic species that can lead to yersiniosis [105,106]. It has been discovered that both *Y. pseudotuberculosis* and *Y. enterocolitica* are capable of causing disease in both humans and animals [107]. Avian pseudotuberculosis is a prevalent disease found worldwide and caused by *Yersinia pseudotuberculosis*. It affects various species of poultry, with young turkeys being the most susceptible. Along with poultry, a diverse range of wild birds and rodents are also prone to this disease [108]. Infection caused by this pathogen is also commonly observed in canaries and wild finches during European winters. The clinical signs include non-specific symptoms such as ruffled feathers, debilitation, and high mortality [109]. This particular pathogen has also been isolated from captive toucans [110], captive pink pigeons [111], black grouse, willow ptarmigan [112], as well as amazon parrots [113], and Eurasian collared doves [114]. A study conducted by Cork et al. in 1999 [115] examined 14 cases of avian pseudotuberculosis. The research revealed that the passerine species demonstrated an acute clinical course of the disease, with predominant enteric bacterial lesions. On the other hand, the pigeons and the psittaciformes examined exhibited a more chronic clinical course of the disease. These findings contribute to the understanding of the diverse manifestations of avian pseudotuberculosis in different avian species.

Recently, a study revealed that a cat was found to have been infected with *Y. pseudotuberculosis*. This particular feline had been diagnosed with diabetes mellitus, acromegaly, and dysorexia. The leading theory is that the cat may have contracted the infection after having contact with natural reservoirs such as rodents or wild birds. This discovery highlights the importance of considering the possibility of transmission to humans, especially when cats share the same household as their owners [116]. In humans, *Y. pseudotuberculosis* not only causes normally self-limiting gastroenteritis but also pseudoappendicitis, arthritis, pharyngitis, and erythema nodosum [117,118,119,120].

*Y. enterocolitica* infection is primarily transmitted to humans through the consumption of contaminated food or water. The pathogenic event commences with bacterial colonization of the intestinal tract, where it causes most of the pathologic effects and clinical manifestations [121]. *Y. enterocolitica* is the most significant cause of yersiniosis in humans in Europe [122]. While this pathogen is widely distributed in nature in aquatic and animal reservoirs, swine are considered a major reservoir for human pathogenic strains [123]. However, some authors suggest that birds may serve as carriers, potential reservoirs, and sources of infection for humans. In 2020, the occurrence of *Y*. *enterocolitica* in the vast majority of migratory game species in Poland was studied. The prevalence of *Y*. *enterocolitica* was determined at 1.4% in green-winged teals, at 5.0% in Eurasian coots, and at 4.8% in capercaillie [124]. This pathogen has also been found in male blackcaps [125], as well as pheasants, crows, magpies, bulbuls, and starlings [126].

Fukushima et al. [127] found that the close relationship between the regional distributions of *Y. pseudotuberculosis* in wild animals and humans suggests that wild animals are an important source of infection, while Niskanen et al. [107] suggest that birds are unlikely to be a direct source of *Yersinia* infections in humans. However, the identification of *Y. pseudotuberculosis* and *Y. enterocolitica* in bird feces indicates that wild birds cannot be excluded from the epidemiological discussion of human yersiniosis.

## 4. Virulence Profiles and Genetic Diversity of Bacteria Isolated from Free-Living Birds

Virulence factors are bacteria-associated molecules that enhance their ability to evade the host defences and cause disease [128]. These factors are either secretory, membrane-associated, or cytosolic [129]. The prevalence of virulence profiles of bacteria is one of the most important issues in recent microbiological studies, and it is worth studying which virulence factors prevail among free-living birds.

Although birds are considered the main reservoir for *Campylobacter* spp., the major cause of gastroenteritis in humans, there is little knowledge about the virulence profiles of *Campylobacter* isolated from free-living birds. Despite the large quantity of research that has revealed the frequency of *Campylobacter* spp., only a few studies have conducted investigations into the virulence genes crucial to pathogenesis [18]. Although the pathogenesis of *Campylobacter* infection is not completely understood, multiple mechanisms are thought to be involved, and virulence factors including adhesion, bacterial invasion, and toxin generation are thought to play a role in its pathogenesis in humans [19,130]. Important virulence factors such as *flaA*, *cadF*, and *ciaB* are necessary for *Campylobacter* to adhere to and colonize epithelial cells in the host intestine [131]. The *cadF,* a gene encoding *Campylobacter* adhesion to fibronectin F [132], *flaA* encoding flagellin A protein, and *ciaB* encoding invasion antigen B have been found in many wild bird species. *FlaA* and *cadF* were present in 100% of *C. jejuni* isolates obtained from free-living birds in China, including crows, Daurian jackdaws, and silver pheasants, with the overall prevalence of this bacteria among wild birds at the level of 10.96% [133]. Similar results were achieved by Shyaka et al. [134], who found *cadF*, *flaA*, and *ciaB* in 100% of *C. jejuni* strains isolated from crows, pigeons, and Eurasian tree sparrows in Japan. Wei et al. [135], in epidemiological studies in South Korea, noticed among *Campylobacter* isolates from wild birds the common prevalence of genes *flaA*, *cadF*, *racR*, and *dnaJ* responsible for colonization and adhesion, genes *ciaB* and *pldA*, which are responsible for invasion, and genes *cdtA*, *cdtB* and *cdtC* encoding cytolethal distending toxin (CDT). Similar findings were confirmed by Shyaka et al. [134] in studies on the characterization of *Campylobacter* isolated from resident wild birds in Japan. *Campylobacter*, specifically *C. jejuni*, can trigger the polyneuropathic disorder denominated Guillain–Barré syndrome (GBS). The *C. jejuni* strains that can elicit GBS carry either *wlaN* or *cgtB*, coding both genes for a β-1,3-galactosyltransferase enzyme that is required to produce sialylated lipooligosaccharide (LOS^SIAL^). In a study by Guirado et al. [136] a similar percentage of strains were positive for LOS^SIAL^-related genes (*wlaN* and *cgtB*) among human (28%) and broiler chicken (22%) strains, while the percentage increased to 40% among wild bird strains. The distribution of the *wlaN* gene ratio shows significant differences ranging from 30% among wild birds in Ireland [137] up to 61.1% in Poland [138]. In comparison, the presence of the *wlaN* gene in poultry varies from 10.7% in Brazil [139] and 13.6% in Germany [140] up to 34.6% in Egypt [141]. The screening of the *cgtB* and the *wlaN* genes in Tunisian poultry revealed the presence of *cgtB* in 21.2% of *C. jejuni* strains, whereas none of them carried the *wlaN* gene [142]. This is concerning, as GBS may be fatal in the acute phase but also affects long-term prognosis due to irreversible sequelae and secondary medical complications [143].

While numerous pathogens are solely taken up by professional phagocytes, *Listeria monocytogenes* may invade nonphagocytic cells such as epithelial cells [144], hepatocytes [145], and endothelial cells [146]. As a result, *L. monocytogenes* may pass through three major barriers: the intestinal epithelial cell barrier, the blood–brain endothelial cell barrier, and the fetoplacental endothelial cell barrier [147]. One of the key virulence factors of *Listeria monocytogenes* is the ability to invade host cells and replicate intracellularly. The internalin (*inl*) gene family, which encodes proteins that interact with host cell receptors to mediate invasion, is one of the virulence genes implicated in this process. The most well-known members of this family, *InlA* and *InlB*, have been demonstrated to be necessary for the pathogenicity of *L. monocytogenes* in animal models [148]. Additionally, *L. monocytogenes* needs the *actA* gene, which encodes a protein involved in actin polymerization and motility, to move intracellularly and spread from one cell to another [149]. In a study performed by Chen [150] in the USA, the common prevalence of *inlA*, *inlB*, and *actA* genes was noted among *L. monocytogenes* strains isolated from wild birds; moreover, these strains were identified as putatively hypervirulent *L. monocytogenes* strains.

Listeriolysin O (LLO) is another major virulence factor in this bacterium, which is encoded by the *hlyA* gene. LLO is required by *L. monocytogenes* for virulence and is found only in virulent strains of the species [151]. Gu et al. [152] described an outbreak of listeriosis among pheasants in China and the *hlyA* gene sequence from the isolated strains showed 99% similarity with several *L. monocytogenes* strains in the GenBank database. According to Hellström et al. [35], *L. monocytogenes* strains are frequently similar to those detected in foods and food processing environments; thus, birds may disseminate *L. monocytogenes* in nature and may also contaminate foods when entering food processing environments and outdoor marketplaces.

Concerning *Clostridium perfringens*, its pathogenicity is primarily caused by the synthesis of various toxins, which are encoded by genes found on plasmids or inside the bacterial chromosome. Many of them have gastrointestinal tract activity, which determines the bacteria’s pathogenicity [153]. The alpha-toxin (*cpa*) gene, which codes for an extracellular phospholipase C, is a significant virulence factor of *C. perfringens* and has been linked to several illnesses, including gas gangrene and food poisoning [154]. Additionally, it has been demonstrated that the beta-toxin gene (*cpb*) encodes a powerful cytotoxin that generates holes in host cell membranes and causes cell death. Moreover, the epsilon-toxin gene (*etx*) gene encodes a necrotizing toxin that causes severe harm to host tissues and contributes to the virulence of *C. perfringens* [155]. In the study conducted in China by Cao et al. [156] among *C. perfringens* strains recovered from wild birds, the prevailing prevalence rate *cpa*, *cpb*, and *etx* genes were noted respectively in 100%, 81.5%, and 44.4% of the isolates. Such common prevalence of genes-affected pathogenic properties is disturbing, as *C. perfringens* strains producing enterotoxin (CPE) are the second most common cause of bacterial food-borne illness in the USA [153].

*Staphylococcus* bacteria are traditionally classified into two groups based on their capacity to coagulate blood plasma (the coagulase response); furthermore, coagulase-negative staphylococci are normally less virulent and express fewer virulence factors [157]. Bacteria in this genus can produce a variety of virulence factors, including surface proteins required for colonization and extracellular toxins responsible for tissue damage and the deactivation of host defence systems [158]. Sulikowska et al. [159] characterized *Staphylococcus* bacteria recovered from dead free-living birds and captive capercaillies kept in south-eastern Poland. The results of multiplex PCR for the five classical enterotoxins A–E showed that the genome of *S. aureus* derived from pheasants, owls, and thrushes contained the gene *sea*, responsible for the production of enterotoxin A. A study performed in Spain on genomic characterization of *S. aureus* derived from wildlife, including birds, revealed the common presence of resistance genes as well as superantigens (such as exfoliative toxins, enterotoxins, or toxic shock syndrome toxin 1) implicated in the pathogenicity of certain human *S. aureus* infections. These findings highlight the role of wild animals as a reservoir for some clinically relevant *S. aureus* strains and the importance of wildlife surveillance, because the environment–animals–humans interaction impacts the transmission and evolution dynamics of *S. aureus* [160].

In different studies, the prevalence of diarrhea-inducing virulence factors among *E. coli* strains derived from free-living birds has been noted. Research conducted in Italy by Bertelloni et al. [79] revealed the high incidence of the *eae* gene (encoding intimin, an outer membrane protein) of EPEC and the *stx* gene (encoding Shiga toxin) of STEC among *E. coli* strains originating from seagulls, waterfowl, and feral pigeons. These birds frequently reach and contaminate both rural and urban areas with their droppings, and may be important sources of *E. coli* infection for other animals and humans. Also, Chandran and Mazumder [161] confirmed the significant distribution of STEC and EPEC strains in a wide range of free-living birds in Canada, with the overall prevalence of the *stx* and *eae* virulence genes noted at mean levels of 23% and 15%, respectively. However, the distribution of these genes differs significantly among bird species; for the *stx* gene, the prevalence rate ranges from 91% in ravens up to 3% in songbirds, and for the *eae* gene, from 93% in hawks up to 4.6% in geese. Interestingly, the studies performed by Seleem et al. [162] covering the phylogenetic analysis of the obtained *stx* genes revealed high genetic relatedness to those isolated from human cases in countries where such birds either lived or which were in their migratory pathway. In conclusion, the study underlines the potential role of migratory birds in spreading STEC across their migratory path.

*Salmonella* is known to possess many virulence genes that play an important role in bacterial pathogenesis, including fimbriae, flagella, or Type III secretion systems (T3SS). These virulence factors allow bacteria to connect to host cells, penetrate tissues, and avoid detection by the host immune system [163,164,165,166]. According to a study by Iwahori et al. [167], *Salmonella* isolates from wild birds in Japan exhibited a comparable distribution of virulence genes to those isolated from other animal species and humans. The genes belonging to T3SS encoded by Salmonella pathogenicity island-1 (SPI-1) and -2 (SPI-2) were found in the majority of the *Salmonella* isolates, as were the fimbriae and flagella genes. Those findings followed studies performed by Matias et al. [168] in Brazil and by Thomas et al. [169] in Oklahoma, which emphasize that the common existence of these virulence genes shows that wild birds may serve as a reservoir for *Salmonella*, which might infect people and other animals.

*Vibrio* spp. recovered from free-living birds are known to possess various virulence traits, which contribute to their pathogenicity. The type III secretion system (T3SS) is one such virulence factor that is involved in the release of effector proteins directly into host cells [170]. It has been shown to contribute to the colonization of the host intestine and the induction of diarrhea [171]. The cholera toxin (CT), which causes severe diarrhea associated with cholera [172], is another key virulence component of *Vibrio* species. Although CT is most often linked with *Vibrio cholerae*, other *Vibrio* species, such as *V. parahaemolyticus*, have been demonstrated to generate a toxin comparable to CT, known as thermostable direct hemolysin (TDH) [173]. TDH has been discovered in certain *V. alginolyticus* isolates, indicating that this virulence factor may also play a role in this species’ pathogenicity [174]. In a study conducted by Zheng et al. [175], it was reported that positive *V. parahaemolyticus* strains were detected in mallard faeces in Japan during the month of February, while in brown-headed gull feces in Thailand, the strains were detected in the warmer months of March, September, and October. The researchers conjectured that the acquisition of virulence genes, specifically TDH, may be unrelated to factors such as season, temperature, bird species, or location. The genome analysis of *V. parahaemolyticus* from aquatic bird feces in Thailand proved that the organization of the T3SS genes in bird fecal isolates was almost identical to those of human clinical strains, posing public health concerns about pathogen dissemination in recreational areas [176]. According to the study, aquatic birds carry a variety of new strains of *V. parahaemolyticus*, which are potentially harmful and found in the marine environment. The genetic diversity of *Vibrio* spp. Strains makes the birds an alternative source of this bacteria.

*Yersinia enterocolitica* is a heterogeneous species divided into six biotypes and various serogroups. The different bioserovars exhibit distinctive virulence properties, hosts, and geographical distributions [177,178]. Pathogenic strains of *Y. enterocolitica* and *Y. pseudotuberculosis* carry chromosomal (*iail*, *invA*, and *ystA*) and plasmid-borne (plasmid of *Yersinia* virulence, pyV) genes, e.g., *yadA* and virF, which are necessary for full virulence [179].

In a study by Niskanen et al. [107], the authors managed to isolate three *virF*-positive *Y. pseudotuberculosis* strains and two *virF*-positive *Y. enterocolitica* strains. The strains of *Y. pseudotuberculosis* came from thrushes, two from song thrushes and one from a redwing. All *Y. pseudotuberculosis* strains were of bioserotype 1/O:2, which has been reported to be associated with human yersiniosis in different countries in Europe [180,181]. Ten of the *Y. enterocolitica* isolates, all isolated from barnacle geese, belonged to bioserotype 3/O:3, which is associated with human disease. Two of these strains were found to be *virF* positive; thus, they carried the virulence plasmid. While the other eight isolates were *virF* negative, they should still be considered to be potentially pathogenic to humans, since the accidental loss of the plasmid during isolation procedures is possible [107]. Odyniec et al. [124] found 1A/NI to be the predominant bioserotype of *Y. enterolocolitica* in wild birds. The presence of single *Y*. *enterocolitica* strains belonging to bioserotypes 1A/O:9 and 1B/NI was also demonstrated. It is noteworthy that in Poland, mallards have yielded five distinct serotyped strains of *Y. enterocolitica*. What piques curiosity about these findings is that three out of the five strains belonged to serotype O:8. Normally, strains that fall under the O:8 serotype are categorized as biotype 1B and are deemed the most harmful to humans [182].

## 5. Antimicrobial Resistance Profiles of Bacteria Isolated from Free-Living Birds

Antimicrobial resistance is a major cause of death worldwide, with the greatest burdens in low-resource settings [183]. This global health challenge involves the transfer of bacteria and genes between humans, animals, and the environment [184]. The acquisition of resistance genes is believed to increase the pathogenicity of microorganisms and the severity of infection with a large possibility of therapy failure [185].

Wild birds have been described as sentinels, reservoirs, and potential antibiotic resistance spreaders [11]. It is crucial to remember that transmission of bacteria, especially antimicrobial-resistant bacteria (ARB), between humans, farm animals, pets, and wild animals is not fully understood. Because of their long-distance mobility, wild birds may transmit ARB over long distances, contributing to the spread of these strains [66,186]. The scale of antimicrobial resistance in wildlife, despite several reports on this subject, continually seems to be incompletely known and underestimated [187,188,189,190]. Although wild birds are infrequently exposed to antimicrobial agents, their faecal waste might function as reservoirs and potential disseminators of resistant bacteria in the environment [4]. It is believed that birds contract resistant bacteria from humans and other sources by consuming infected food or water [3]. Antibiotic residues and bacteria expressing antibiotic resistance may enter the environment as a result of the spread of medicated animal manure and urban effluents into agricultural land [191]. Environmental pollution, the presence of livestock, and human density are all variables that also contribute to the incidence of resistant bacterial strains in wild birds [192,193].

One of the most significant zoonotic pathogens linked to multidrug resistance illnesses in people and birds is *Campylobacter* spp. [194]. Migratory wild birds may have a substantial impact on the spread of *Campylobacter* spp. to farm animals and their habitats, particularly poultry farms, as these bacteria reside in the digestive tracts of wild birds [195,196,197,198]. *Campylobacter* has developed several resistance mechanisms in response to the use of antibiotics in clinical settings and animal agriculture. As a result, antibiotic-resistant *Campylobacter* is becoming more common, endangering the efficacy of antibiotic treatments, and raising serious concerns for public health [199]. In the treatment of human campylobacteriosis, fluoroquinolones and macrolides are commonly considered the first line of defence [200]. The antibiotic resistance data observed in wild birds in connection with the aforementioned treatment approach are alarming. According to Casalino et al. [201], most *Campylobacter* isolates from wild birds show resistance to trimethoprim (52.1%), ciprofloxacin (43.7%), and enrofloxacin (31.2%), while Indykiewicz et al. [202] state that *Campylobacter* spp. are commonly susceptible to azithromycin (97.62%) and erythromycin (95.24%), and slightly less frequently to tetracycline (50.0%) and ciprofloxacin (47.62%). The multidrug resistance of *Campylobacter* strains varies from 21.56% in Italy [201] up to 72% in Egypt [198]. The findings of the study indicate that wild birds can serve as carriers of antimicrobial-resistant *Campylobacter* spp. strains, posing a significant threat to the well-being of both animals and humans. Extended and unwarranted utilization of antibiotics has led to the development of resistance in *Campylobacter*, thereby making the treatment of human infections caused by this bacterium increasingly challenging [203]. The discovery of innovative treatments is often a formidable task, particularly when contending with Gram-negative bacteria that have evolved an outer membrane as a defence mechanism against undesirable compounds. Consequently, finding new substances that can effectively combat these bacteria presents a significant challenge [204].

The capacity of *Listeria* species to quickly acquire resistance to any antimicrobial treatment, as has been shown with other infections important to humans, poses a new and growing hazard to human and animal health [205]. It mostly affects high-risk patient populations, where it can result in serious and fatal infections [206,207,208,209]. As a result, antibiotic therapy is frequently required to manage the illness caused by this bacterium [210]. Ampicillin, either alone or in combination with gentamicin, is the current therapy strategy for severe cases of listeriosis [211]. At present, our knowledge regarding the antibiotic resistance of Listeria spp. in wild birds is limited. Jagtap et al. [212] found the isolates gained from wild birds to be susceptible to all antibiotics when subjected to testing, while Fadel et al. [213] were not able to perform AMR testing, as *L. monocytogenes* isolates lost their viability during preservation. The data currently accessible pertain predominantly to farm-raised poultry. Based on the results published by Cokal et al. [214], it has been determined that 6.7% of the samples taken from both broiler and layer flocks exhibited resistance to both ampicillin and gentamicin, respectively. Additionally, findings indicated that the presence of multidrug-resistant (MDR) isolates was observed in 20% of the samples. It can be postulated that the prevalence of AMR in *L. monocytogenes* in the environment may lead to comparable findings in birds that are not under human care. Nonetheless, additional research is imperative to establish the extent of antibiotic resistance among these avian species.

At present, there exists a paucity of available data regarding the antimicrobial resistance exhibited by *C. perfringens* strains isolated from wild avian populations. Previous research has focused predominantly on assessing the susceptibility of livestock species to this pathogen. Antimicrobial susceptibility testing of ducks in China [48] showed that isolates demonstrated the highest resistance against gentamicin (95.72%), followed by bacitracin (71.05%) and lincomycin (65.79%). Despite the relatively high degree of antimicrobial resistance exhibited by these antibiotics, further data have revealed an even more concerning situation. In human medicine, penicillin and clindamycin are the preferred antibiotics for treating infections caused by *C. perfringens* [215], while cephalosporins are considered alternative drugs for penicillin-allergic patients [216]. It is worrisome to note that isolates showed resistance to penicillin (7.24%), cefotaxime (2.96%), and cefepime (19.08%). A significant proportion, 81.58%, of the isolates obtained have been identified as multi-drug resistant. This raises concerns about the effectiveness of current treatment regimens and underscores the need for urgent action.

It is pertinent to note that the *mecA* gene, responsible for encoding the penicillin-binding proteins that render beta-lactam antibiotics ineffective, is the principal cause of methicillin resistance in *Staphylococcus aureus*. This protein has enabled *Staphylococcus aureus* to evolve robust resistance mechanisms against clinically vital antibiotic classes, i.e., penicillins and glycopeptides, that are the treatment of choice in Staphylococcal infections [217]. A study by Kutkowska et al. [218] suggests that rooks from urban areas and passerine birds from the natural habitat carry antibiotic-resistant *S. aureus* strains, probably reflecting the presence of such isolates in environmental food sources. Their tests revealed that 19.5% of *S. aureus* isolates from rook samples and 37.5% from wild-living birds were resistant to methicillin. The strains of *S. aureus* isolated from free-living birds in southeastern Poland exhibited resistance to enrofloxacin (44.2%), tetracycline (36.4%), fusidic acid (36.4%), erythromycin (33.3%), and ampicillin (33.3%). In addition, more than 20% of staphylococcal strains were resistant to three additional antibiotics: penicillin G, clindamycin, and amoxicillin [159]. In a study conducted by Russo et al. in 2022 [3], it was found that all isolates were resistant to clindamycin and cefoxitin, while 66.6% were also resistant to erythromycin, doxycycline, tetracycline, and trimethoprim–sulfamethoxazole. The clinical community recognizes resistance to beta-lactam antibiotics (penicillins, cephalosporins, monobactams, and carbapenems) as the most perilous, due to their highly effective and wide-ranging mechanism of action against Staphylococcal infections. Furthermore, these drugs exhibit low toxicity in both humans and animals, further enhancing their therapeutic value [219].

Antimicrobial resistance in *E. coli* poses a public health concern worldwide [76]. This pathogen has been linked to systemic illness in birds and is the most common opportunistic enterobacteria in captive animals [80,220]. Shobrak et al. [221] captured six types of migrating wild birds and nine types of non-migrating birds from the Taif area for isolation of *Escherichia* spp. The prevalence range varied from 92% in non-migrating birds to 94% in migrating birds. All isolates recovered from non-migrating birds were found to be resistant to oxacillin, while all isolates recovered from migrating birds demonstrated resistance to oxacillin, chloramphenicol, oxytetracycline, and lincomycin. In the study, all examined isolates exhibited multidrug-resistant (MDR) phenotypes that were resistant to approximately 3–10 antimicrobial agents. The authors of the study posit that wild birds may have been exposed to antimicrobial residues and bacteria that had developed antibiotic resistance as a result of human waste in waterways. Additionally, certain antimicrobial agents employed in human and veterinary medicine may not be fully eliminated, resulting in their dispersion in the environment via wastewater and soil [222]. Hasan et al. [223] discovered that *E. coli* isolated from both domestic and wild birds was most commonly resistant to tetracycline, ampicillin, trimethoprim/sulfamethoxazole, and nalidixic acid, while Nowaczek et al. [224] found *E. coli* isolated from free-living birds to be resistant to gentamicin too. On average, MDR was found in 42.7% of the isolates obtained from avian samples [223,224,225,226]. In the treatment of infections caused by *E. coli*, such as those acquired in the community or a hospital setting, the typical approach has been to use first-line antibiotics. These include cephalosporins, fluoroquinolones, and trimethoprim–sulfamethoxazole. However, managing *E. coli* infections is becoming increasingly complex due to the emergence and spread of multiresistance to these commonly used antimicrobial agents [227]. The global prevalence of antibiotic resistance in *E. coli* over the years has been a cause for concern and emphasizes the importance of appropriate measures to prevent transmission [228].

*Salmonella* is widely distributed among birds, making them an excellent indication of the AMR issue affecting wildlife [229]. According to a recent study conducted in Spain, a substantial proportion of *Salmonella* strains isolated from wild birds demonstrated resistance to at least one of the twelve antimicrobials tested. Specifically, the study revealed that 40.5% of the strains analyzed exhibited such resistance to ciprofloxacin (29.7%), nalidixic acid (29.7%), ampicillin (10.8%), colistin (21.6%), or tetracycline (13.5%) [229]. Ciprofloxacin, nalidixic acid, and colistin are antimicrobial agents of last resort that are utilized in the treatment of human infectious diseases caused by multi-resistant bacteria. These drugs are typically employed in the context of severe, life-threatening infections [230,231]. Due to the elevated levels of antimicrobial resistance observed in these particular antibiotics, there must be ongoing and vigilant monitoring of AMR in wild bird populations. This highlights the necessity for continued surveillance of wild bird populations to identify and track AMR trends. Different antibiotic sensitivity patterns were observed by Spinu et al. [232]. They discovered that 100% of *Salmonella* spp. isolates from birds in the Danube Delta were resistant to penicillin, amikacin, and erythromycin. Wild birds in Turkey carried *Salmonella* serotypes that were all resistant to lincomycin, nalidixic acid, and penicillin [233], whereas in Italy, almost all the analyzed strains (97.7%) showed resistance to at least one class of antibiotics, with the highest resistance values observed for tetracycline (81.8%) [234]. The research findings underscore the importance of maintaining a vigilant approach to monitoring antimicrobial resistance in wild bird populations and implementing effective strategies to mitigate this issue.

The incidence of drug-resistant strains of *Vibrio* spp. has been observed to be on the rise [235,236]. Out of the 76 *Vibrio* spp. isolates obtained from migratory and sedentary birds in Romania, 81.57% showed a multidrug resistance phenotype. Additionally, 18.42% of the strains were resistant to at least three antimicrobials, which mainly included penicillin, aminoglycosides, and macrolides [101]. Aminoglycoside antibiotics (AGAs) represent a category of cationic antibiotics that exhibit broad-spectrum activity. They are widely employed for the treatment of Gram-negative bacterial infections. However, the increasing prevalence of bacterial resistance to AGAs is leading to suboptimal treatment outcomes. Consequently, the efficacy of AGAs as a treatment option for Gram-negative bacterial infections is being eroded [237]. Antibiotic sensitivity experiments performed in China revealed that 37.10% of isolates obtained from migratory birds were resistant to ampicillin [175]. The rise in the occurrence of multidrug-resistant strains of *V. cholerae* on a global scale is a matter of growing concern. This trend not only poses a significant challenge to public health but also underscores the need for enhanced surveillance and control measures. Given the severe consequences of cholera outbreaks, particularly in resource-limited settings, it is critical that we take proactive steps to address this emerging threat.

An area of particular concern within the field of antimicrobial resistance research pertains to the dearth of comprehensive data regarding the susceptibility patterns exhibited by Yersinia spp. strains isolated from avian hosts. Despite the critical importance of understanding the antimicrobial resistance profiles of these pathogens, the existing body of literature is notably sparse in this regard, underscoring a significant gap in our current understanding of the potential transmission dynamics and selective pressures contributing to antimicrobial resistance within avian populations. The analysis of antimicrobial susceptibility in *Y. enterocollitica* from wild bird strains revealed that all tested isolates were resistant to amoxicillin with clavulanic acid, ampicillin, and cefalexin. It is noteworthy that the strains isolated from migratory birds such as green-winged teals and Eurasian coots, and those obtained from birds living in a specific territory like capercaillie, showed different levels of resistance to chemotherapeutics. The strains isolated from migratory birds were also resistant to kanamycin and streptomycin, and they were resistant and intermediately resistant to cefotaxime, ceftazidime, chloramphenicol, gentamycin, and tetracycline, to which the strains isolated from the capercaillie were susceptible. Susceptibility to only one or two chemotherapeutics is a cause for concern, as migratory birds rely on public sources of drinking water [124].

## 6. Conclusions

The findings of this systematic literature review shed light on the intricate interplay between free-living birds and pathogenic bacteria, emphasizing the significant public health implications inherent in understanding and addressing this dynamic relationship. The movement of birds across vast geographical areas, coupled with environmental stressors and human interaction, amplifies the potential for the transmission of bacterial pathogens to both avian and human populations.

The review underscores the global nature of the transmission dynamics of bacterial pathogens carried by free-living birds. The migratory patterns of avian species facilitate the dissemination of infectious agents across continents, transcending geographical boundaries and posing challenges to traditional disease surveillance and control measures. In light of increasing urbanization and the encroachment of human settlements into avian habitats, the risks associated with zoonotic pathogens carried by birds are further compounded.

Multidisciplinary research efforts are essential to elucidate the epidemiological dynamics of pathogenic bacteria in free-living birds and their implications for public health. Collaboration among ornithologists, microbiologists, epidemiologists, and public health officials is imperative to enhance surveillance, early detection, and mitigation strategies for zoonotic disease transmission. By fostering interdisciplinary collaborations, researchers can gain a comprehensive understanding of the complex interactions between avian species and bacterial pathogens, thus informing evidence-based interventions to safeguard public health [238].

Public awareness initiatives play a pivotal role in promoting proactive measures to minimize the transmission of bacterial pathogens from free-living birds to humans. Educating the public about the potential risks associated with interactions between humans and birds can empower individuals to adopt preventive measures and mitigate the spread of zoonotic diseases. Moreover, targeted communication strategies can help dispel misconceptions and promote responsible behaviors concerning bird–human interactions.

In conclusion, by comprehensively understanding the epidemiological significance of avian species in transmitting bacterial pathogens, proactive measures can be implemented to mitigate the risks posed to both human and animal populations. Through sustained research efforts, collaborative partnerships, and public engagement, we can effectively address the challenges posed by the transmission of bacterial pathogens by free-living birds, thereby safeguarding public health in an era marked by increasing urbanization and the emergence of zoonotic illnesses.

## Data Availability

Not applicable.
